# ﻿*Rubusdianchuanensis* sp. nov. (Rosaceae) from Sichuan and Yunnan, southwest China

**DOI:** 10.3897/phytokeys.193.82287

**Published:** 2022-04-01

**Authors:** Qiu-Ping Wang, Yu-Ran Li, Qiang-Chun Huang, Huan-Chong Wang

**Affiliations:** 1 School of Ecology and Environmental Science, Yunnan University, Kunming 650500, Yunnan, China Yunnan Univerisy Kunming China; 2 School of Life Sciences, Yunnan University, Kunming 650500, Yunnan, China Yunnan University Kunming China; 3 Herbarium of Yunnan University, Kunming 650091, Yunnan, China Yunnan Univerisity Kunming China

**Keywords:** Endemism, later homonym, *Rubussubornatus*, synonymy, taxonomy

## Abstract

*Rubusdianchuanensis*, a new name for the species previously named as *R.vicarius* by W. O. Focke in 1911, is proposed. A detailed description, illustrations, and remarks on its phenology, ecology, and geographic distribution are provided. This raspberry (subg. Idaeobatus) is endemic to China and was only found in Sichuan and Yunnan, southwest China. Morphologically, it is most similar to *R.ovatisepalus* but clearly differs from the latter by having leaf densely white or grey tomentose abaxially, usually leaf-like bracts at the base of inflorescence, 1–6 cm long pedicels, and triangular-ovate sepals with acute to caudate apex.

## ﻿Introduction

The genus *Rubus* was established by Linnaeus (1753) in his *Species Plantarum* and ten species were described there. Currently, about 700 species of *Rubus* are recognized, making it one of the largest genera of Rosaceae (Robertson 1974; Lu and Boufford 2003). Plants of this genus are usually shrubs, rarely subshrubs or perennial herbs, and more or less prickly. Their leaves are compound or simple, flowers are pentamerous and mostly bisexual, fruits are fleshy aggregates of drupelets, and tori are usually convex, conical or cylindrical (Kalkman 1993; Lu and Boufford 2003; Wang and Wang 2019).

Members of *Rubus* are distributed nearly worldwide except for Antarctica and can be found in most types of land biomes from tropical to subarctic regions (Gustafsson 1942; Spies and Du Plessis 1984; Hummer 1996; Lu and Boufford 2003). There are more than 250 species of *Rubus* in East Asia, and this region is the center of diversity for the subgenera *Malachobatus* and *Idaeobatus* (Wang and Wang 2019). More than 200 species are recorded in China, and most of them occur in the southern and southwestern provinces (Lu and Boufford 2003). Recently, new species and nomenclatural changes of *Rubus* in China have been constantly reported (e.g., Huang and Hu 2009; Byalt 2011; Sun and Boufford 2012; Wang et al. 2013, 2017, 2019; Wang and Wang 2019).

During our fieldwork and the herbarium studies on a taxonomic revision for the Chinese species of *Rubus*, we encountered a raspberry difficult to assign to any species recognized by Yu and Lu (1985) and Lu and Boufford (2003). Further research showed that it should be identified as *R.vicarius* Focke, which had been synonymized with *R.subornatus* Focke previously (Yu and Lu 1985; Lu and Boufford 2003). This plant represents a separate species, therefore, should be resurrected. Nevertheless, Focke’s name is a later homonym of *R.vicarius*Sudre (1902); consequently, a new name for this distinctive species is required.

## ﻿Materials and methods

We studied the newly named species both in the field and the herbaria. Type specimens (or type photos) of accepted names and their synonyms in Rubussubg.Idaeobatus were extensively examined and compared, as well as herbarium materials from CDBI, IBSC, KUN, P, PE, PYU and YUKU (acronyms after Thiers 2022). Pertinent taxonomic literature (e.g., Focke 1877, 1910, 1911, 1914; Yu and Lu 1985; Lu and Boufford 2003) were extensively consulted. Morphological studies were carried out on dried material under a stereomicroscope, and measurements were conducted using a ruler or a metric vernier calliper.

## ﻿Taxonomy

### ﻿ Rubus dianchuanensis

Taxon classificationPlantae

Huan C. Wang & Q. P. Wang sp. nov.

AEDC4B12-81B6-52A6-BCEC-948678DEBACD

urn:lsid:ipni.org:names:77296909-1

Figs 1 , 2 , 3A1–A5

#### Type.

China. Sichuan Province: Liangshan Prefecture, Muli County, on the way from Wujiao to Yiji, 27°58'21.73"N, 100°41'51.20"E, 3300–3500 m a.s.l., 23 July 2021, *Q. P. Wang et al.ML12992* (holotype YUKU!, isotypes YUKU!).

**Figure 1.**
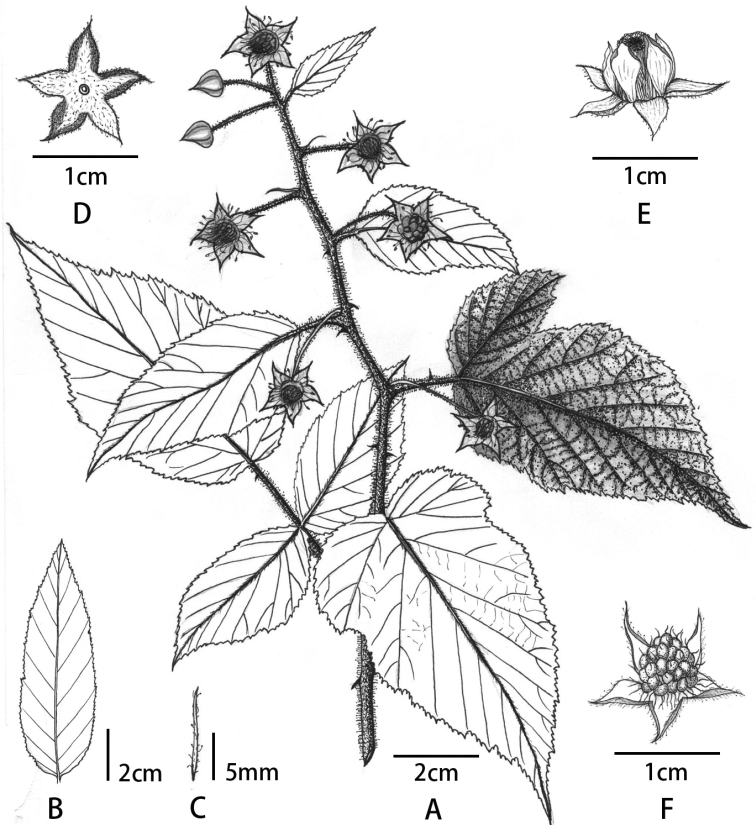
*Rubusdianchuanensis***A** habit **B** bract at the base of inflorescence **C** bract at the upper part of inflorescence **D** calyx **E** flower (side view) **F** aggregate fruit with persistent calyx.

*Rubusvicarius* Focke in Sargent, Pl. Wils. 1: 56. 1911, *nom. illeg.*, non Sudre (1902: 12). Type: China. Sichuan Province, Leshan City, Wa Shan, in thickets, 1500–2100 m a.s.l., July to August 1908, *E. H. Wilson 948* (BM!, NYBG!, US!).

**Figure 2.**
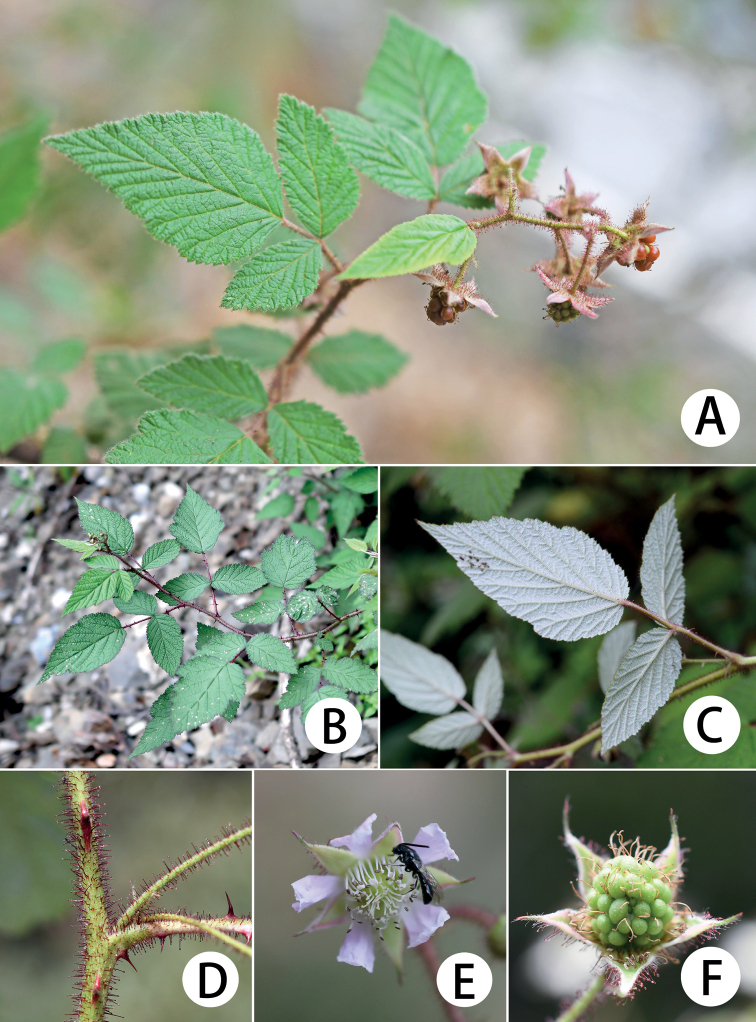
*Rubusdianchuanensis***A, B** habit **C** abaxial surfaces of leaves **D** portion of branchlet showing glandular hairs **E** flower **F** an immature aggregate fruit with calyx.

#### Diagnosis.

*Rubusdianchuanensis* is most similar to *R.ovatisepalus* Huan C. Wang, but clearly differs from the latter by its leaf abaxially densely white or grey tomentose, bracts in the inflorescence often leaf-like, pedicels 1–6 cm long, sepals triangular-ovate and with acute to caudate apex.

**Figure 3.**
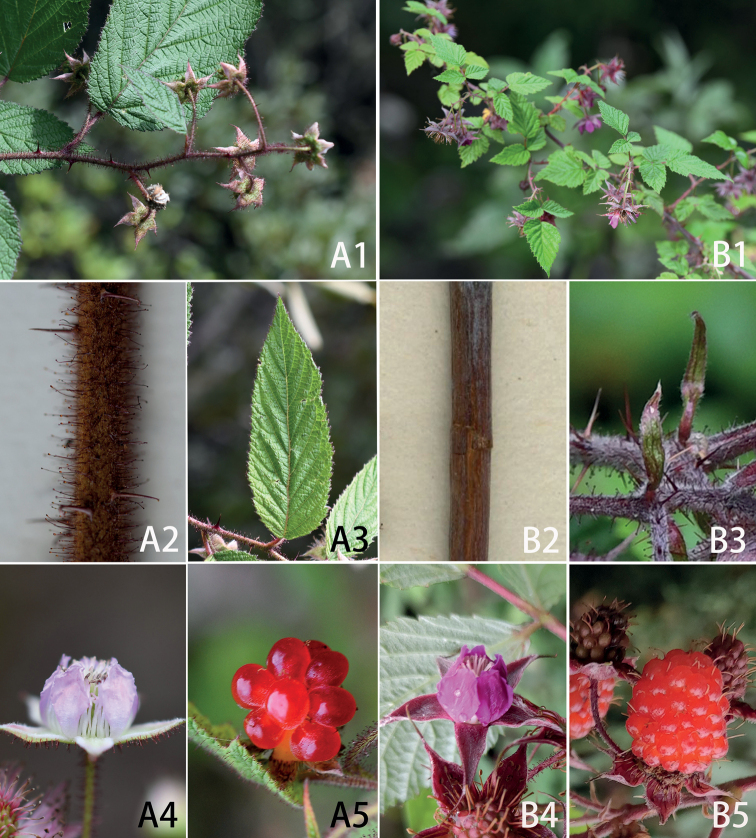
*Rubusdianchuanensis* (**A1–A5**) and *R.subornatus* (**B1–B5**) **A1** a flowering branch showing terminal inflorescence **A2** portion of stem showing indumentum **A3** bract at the base of inflorescence **A4** flower (side view) **A5** mature aggregate fruit **B1** habit **B2** portion of the stem **B3** bracts **B4** flower (side view) **B5** mature aggregate fruit.

#### Description.

Arching shrubs, 1 to 2 m high, deciduous or semi-evergreen. Stems with dense ferruginous glandular hairs and soft eglandular hairs. Branchlets cylindric, grey-green to brown, villous, with curved prickles and nearly straight, ferruginous, 1–2 mm long, glandular hairs. Leaves imparipinnate, usually 3-foliolate, rarely 5-foliolate. Stipules persistent, linear, 5–8 mm long, ca. 1.5 mm wide, pubescent, with glandular hairs, base shortly adnate to petioles. Petioles 0.5–4 cm long, petiolule of terminal leaflets 1–3.5 cm long, lateral leaflets sessile or subsessile; petiolule and rachis with glandular hairs, intermixed pubescence and erect or recurved prickles. Leaf blades cordate or ovate-cordate in outline, papery, adaxially pubescent and with sparse glandular hairs, abaxially densely white or grey tomentose throughout, with sparse glandular hairs along veins. Terminal leaflets cordate, broadly ovate or ovate, 3–11 cm long, 2–7 cm wide, apex acute to acuminate, base rounded to subcordate; margin slightly lobed or not, double serrate; nervation pinnate, with 6–9 lateral veins on each side of the midrib. Lateral leaflets ovate or elliptic, apex acute, base cuneate to round, slightly oblique, 1.5–7 cm long, 1–5 cm wide, lateral veins 5–7 paired. Terminal inflorescences racemose-cymose, 4–10-flowered, 5–15 cm long; bracts at the base usually leaf-like, simple, ovate, ovate-lanceolate or lanceolate, with similar indumentum as the leaves, 2.5–11 cm long, 1–5 cm wide, apex acute to acuminate, base rounded to subcordate; bracts at the upper portion linear, 4–12 mm long, ca. 1 mm wide, pubescent, with glandular hairs. Axillary flowers usually solitary, rarely 2–3-flowered. Pedicels 1–6 cm in length, densely pilose, with dense glandular hairs and curved prickles. Flowers 1–1.5 cm in diameter. Calyx grey-green or reddish, abaxially with soft hairs and glandular hairs; sepals triangular-ovate, erect or spreading after anthesis, 5–10 mm long, 2–4 mm wide, margin grey tomentose and entire, apex acute to caudate. Petals pink to white, obovate, 5–8 mm long, 4–5 mm broad, apex repand, base shortly clawed. Stamens numerous in 2 whorls; filaments linear, glabrous, ca. 5 mm long. Pistils numerous; ovaries sparely pilose, styles glabrous. Aggregate fruit ovoid, orange-red to red.

#### Taxonomic notes.

*Rubusdianchuanensis* was firstly collected by Ernest Henry Wilson in 1908 from Wa Shan (Leshan City) in western Sichuan, southwest China. Based on Wilson’s collection, Focke (1911) published “*R.vicarius* n. form. (?)” with a Latin description in his monograph *Species Ruborum*. However, the name *R.vicarius* Focke was not validly published there under Article 36.1 of the Shenzhen Code (Turland et al. 2018). Shortly afterwards, in July 1911, the name *R.vicarius* Focke was definitely accepted by Focke (in Sargent 1911) and accompanied by a complete and direct reference, namely “Bibl. Bot. LXXII 211 (Spec. Rub.) (1911)”, to his previous Latin description, it was therefore validated. Unfortunately, the name *R.vicarius* had been previously used by Sudre (1902) for a European species; thus, Focke’s name as a later homonym was illegitimate (Article 53.1 of the Shenzhen Code).

**Figure 4.**
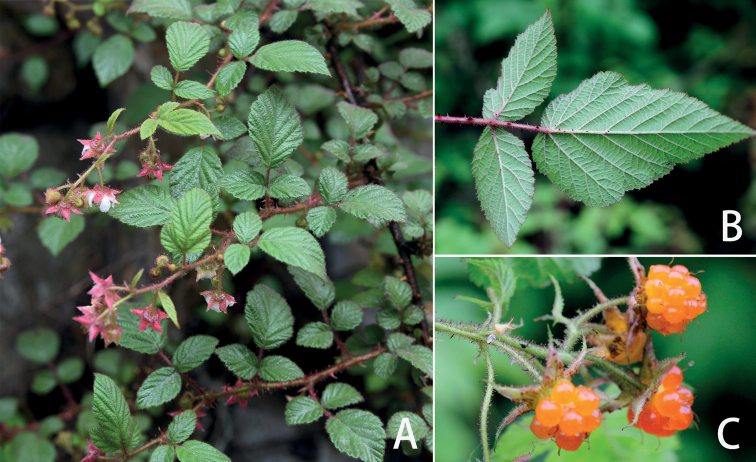
*Rubusovatisepalus***A** habit **B** abaxial surfaces of leaflets **C** mature aggregate fruit.

Morphologically, *Rubusdianchuanensis* is most similar to *R.ovatisepalus* Huan C. Wang (Fig. 4), a species described recently from northwestern Yunnan and southeastern Xizang, southwest China (Wang and Wang 2019), in having dense glandular hairs throughout the plant and the racemose-cymose terminal inflorescences. However, *R.dianchuanensis* differs markedly from the latter by its leaf abaxially densely white or grey tomentose (*vs.* sparsely pubescent, with glandular hairs), bracts at base of the inflorescence usually leaf-like, ovate, ovate-lanceolate or lanceolate (*vs.* lanceolate to linear), 2.5–11×1–5 cm (*vs.* 0.7–1.2×0.1–0.2 cm), flower usually larger, 1–1.5 cm (*vs.* 0.8–1.2 cm) in diameter, pedicels 1–6 cm (*vs.* 0.7–1.5 cm) long, apex of sepals acute to caudate (*vs.* long acuminate to caudate).

*Rubusdianchuanensis* is also similar to *R.subornatus* Focke (including its variety R.subornatusvar.melanodenus Focke) (Fig. 3B1–B5), with which *R.vicarius* Focke had been erroneously synonymized by Yu and Lu (1985) as well as Lu and Boufford (2003). Nevertheless, *R.dianchuanensis* differs from it by its not glaucous stems usually covered with dense ferruginous glandular hairs and soft eglandular hairs (*vs.* more or less glaucous, glabrous), terminal inflorescences racemose-cymose (*vs.* corymbose), bracts at base of the inflorescence often leaf-like, rarely trifoliolate, ovate, ovate-lanceolate or lanceolate (*vs.* linear) (Fig. 3: A3, B3), pedicels usually longer, 1–6 cm (*vs.* 1–2.5 cm) long, calyx abaxially with grey pubescent (*vs.* intermixed tomentose) and dense ferruginous glandular hairs (*vs.* spare or not), without needle-like prickles (*vs.* with needle-like prickles), and slightly pink to white (*vs.* purplish-red) petals. Some specimens of *R.dianchuanensis* had been identified as *R.phoenicolasius* Maxim., but it is well differentiated from the latter by stems and branches with short glandular hairs (*vs.* long glandular hairs) and without bristles (vs. dense bristles), terminal inflorescence racemose-cymose (*vs.* short racemes) 5–15 cm (*vs.* 1–6 cm) long, pedicels 1–6 cm (*vs.* 0.5–1.5 cm) long, flowers 1–1.5 cm (*vs.* 0.6–1.5 cm) in diameter, calyx without bristles (*vs.* with dense bristles), sepals triangular-ovate (*vs.* lanceolate). A detailed morphological comparison between these four species is summarized in Table 1.

**Table 1.** A morphological comparison of *Rubusdianchuanensis* with its relatives.

	*R.dianchuanensis*	*R.ovatisepalus*	*R.subornatus*	*R.phoenicolasius*
**Indumentum of stems**	dense glandular hairs	dense glandular hairs	glabrous	dense glandular hairs and bristles
**Abaxial indumentum of leaf blade**	densely grey tomentose	sparsely pubescent, with glandular hairs	densely grey tomentose	densely grey tomentose
**Terminal Inflorescence**	racemose-cymose	racemiform cymes	corymbose	short racemes
**Length of pedicel**	1–6 cm	0.7–1.5 cm	1–2.5 cm	0.5–1.5 cm
**Diameter of flower**	1–1.5 cm	0.8–1.2 cm	2–3 cm	0.6–1.5 cm
**Petal colour**	white or slightly pink	white or slightly pink	purplish-red	white
**Petal *vs.* sepal**	petal slightly longer than sepals	petal shorter than sepals	petal shorter than sepals	petal much shorter than sepals

#### Phenology.

*Rubusdianchuanensis* flowering from June to August, fruiting from July to September.

#### Etymology.

The specific epithet “dianchuanensis” refers to the Yunnan (called *dian* for short in Chinese) and Sichuan (called *chuan* for short in Chinese) provinces, where this species occurs.

#### Distribution and habitat.

**Figure 5.**
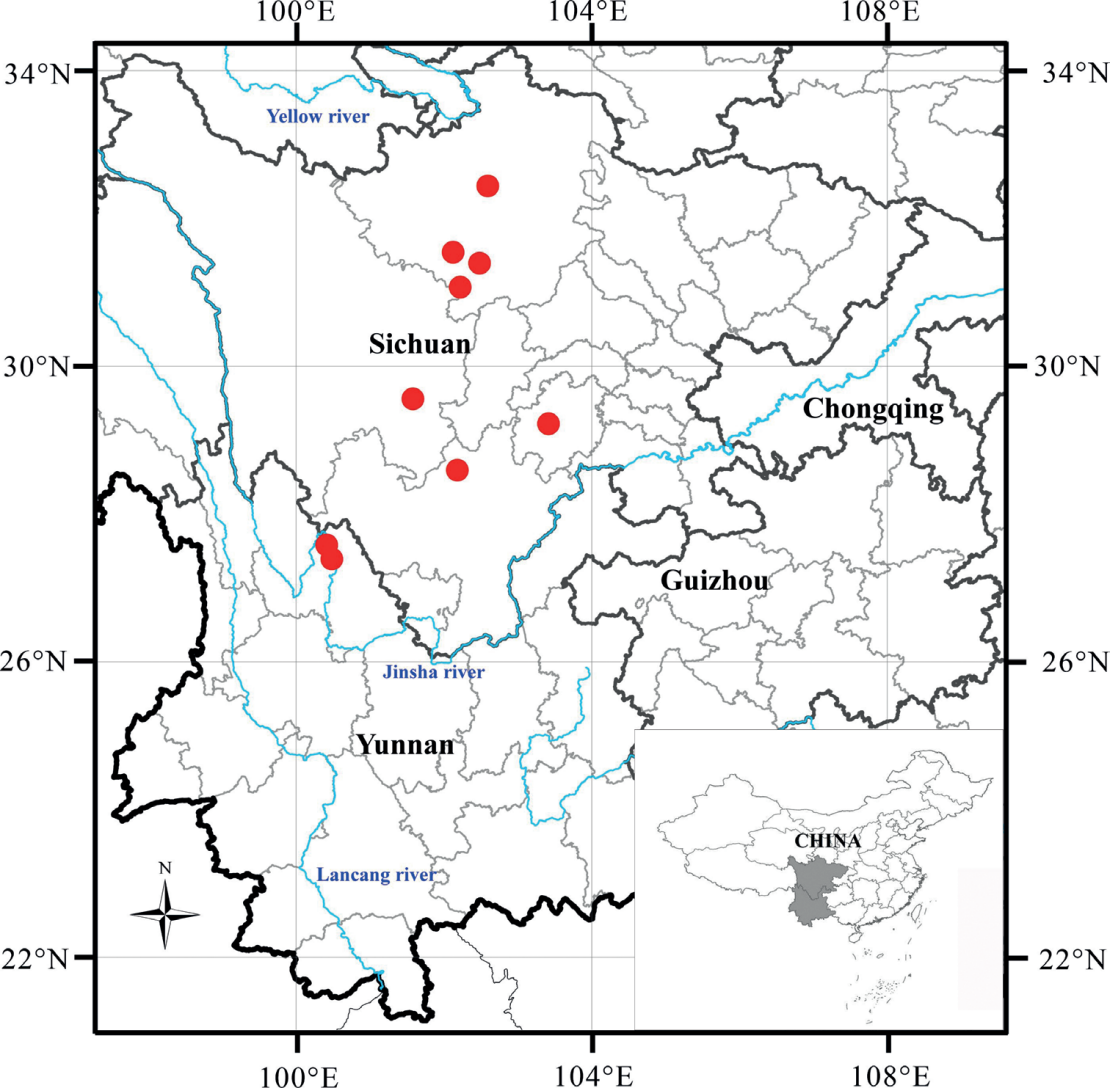
Geographical distribution of *Rubusdianchuanensis* (red dots).

*Rubusdianchuanensis* is endemic to southwest China, where it has been collected from western Sichuan and northwestern Yunnan (Fig. 5). It usually occurs at elevations ranging from 2500–3600 meters and grows in open woods and thickets.

#### Additional specimens examined

**(*Paratypes*)**: China. **Sichuan**: Muli County, Damadian, 3000 m a.s.l., 16 Aug 1937, *T. T. Yu 7740* (PE); Shimian County, Liziping Country, 2700 m a.s.l., 28 Jul 1978, *Shimian Team 78-0875* (SM); Shimian County, 1955, *C. C. Hsieh 41198* (IBSC, PE); Kangding City, Zheduotang village, 3100 m a.s.l., 1 Aug 1963, *K. C. Kuan et all 1218* (PE); same location, 3450 m a.s.l., 5 July 1953, *X. L. Jiang 36185* (IBK, IBSC, PE); same location, 3600 m a.s.l., 16 Jul 1953, *W. P. Fang & X. L. Jiang 36323* (IBK, ISBC, PE); Kangding County, 2750 m a.s.l., 24 Jun 1980, *Z. J. Zhao 112962* (CDBI) and *Z. J. Zhao 119262* (PE); Kangding City, Yajiageng, Laoyunshachang, 3318 m a.s.l., 101°58'17"E, 29°56'00"N, 28 Aug 2008, *Y. L. Peng & W. G. Tu Gaoxf-0856* (KUN); Xiaojin County, 3500 m a.s.l., 1 Jul 1959, *Z. G. Liu 0412* (CDBI, PE); Lixian County, 26 Aug 1957, *X. Li 74160* (IBSC, PE, NAS); Lixian County, Miyaluo village, 25 Jul 1958, *Z. L. Wu 33375* (PE; CAF); Barkam City, Barkam County, Dalangjiao River, 2300–2900 m a.s.l., 12 Jul 1960, *Sichuan Medicine Source Survey Team 22297* (NAS, SM); Barkam City, 2800 m a.s.l., 11 Jul 1957, *H. F. Zhou & Z. Y. Zhang 22772* (IBSC, NAS, KUN, PE); Barkam City, Dalangzugou, 2700 m a.s.l., 27 Aug 1957, *X. Li 72288* (IBSC, NAS, PE); Heishui County, Naizigou, 2900 m a.s.l., 22 Jul 1957, *X. Li 73260* (IBSC, NAS, KUN; PE). **Yunnan**: Ninglang County, Lugu Lake, 27°39'21"N, 100°48'36"E, 2500–2600 m a.s.l., 6 Aug 2015, *H. C. Wang et al. LGH8164* (YUKU).

## Supplementary Material

XML Treatment for Rubus dianchuanensis
